# The Effect of Age on Corneal Topographic Indices, Keratometry and Visual Acuity After Combined Intrastromal Corneal Ring Segment (ICRS) Implantation and Corneal Crosslinking

**Published:** 2020-03-30

**Authors:** Sylvain El-Khoury, Youssef Abdelmassih, Mazen Amro, Ali Dirani, Carol Cherfan, Elias Jarade

**Affiliations:** 1 Beirut Eye Specialist Hospital, Beirut, Lebanon.; 2 Fondation Ophtalmologique Rothschild, Department of Pediatrics and Retina, Paris, France.; 3 School of Medical Sciences, Lebanese University, Beirut, Lebanon.; 4 Mediclinic Dubai Mall, Dubai, United Arab Emirates.; * Authors contributed equally

**Keywords:** Keratoconus, Pediatric Keratoconus, Intracorneal Ring Segment, Crosslinking

## Abstract

We aimed to assess age-related changes in corneal topographic indices, keratometry and visual acuity after sequential intracorneal ring segment implantation (ICRS) and crosslinking (CXL). This was a retrospective matched case-control series including 26 eyes of patients ≤18 years as cases and 26 eyes of adult patients as controls. All eyes received ICRS+CXL for progressive keratoconus. Eyes were matched regarding the keratoconus parameters and the treatment (type, number and thickness of ICRS). Data was analyzed for refractive and topographic values (uncorrected and corrected distance visual acuity (UDVA; CDVA) sphere; cylinder; spherical equivalent; maximum keratometry (Kmax); flat keratometry (Kflat); steep keratometry (Ksteep); all 7 pentacam topographic indices) preoperatively and one year postoperatively. Preoperatively, there was no significant difference for any refractive, clinical or topographic parameters between the groups except for index of vertical asymmetry. After one year, children had a significantly higher improvement in Ksteep (3.05D) than adults (2.10D; P=0.036) and a trend to significance for Kflat (2.7D compared to 1.78D, respectively; P=0.081). UDVA improved by 4.3 ETDRS lines in children compared to 3.3 ETDRS lines in adults and CDVA improved by 1.7 ETDRS lines in children compared to 1.2 ETDRS lines in adults, but with no statistical significance. The effects on keratometry indicated that corneal response after ICRS and CXL for keratoconus is more pronounced in young patients than adults. This assumption is also supported by functional improvement and by the fact that a few eyes (5) of some very young patients (<13years) showed highly remarkable improvements after surgery (higher than any adult eye).

## INTRODUCTION

Keratoconus is a progressive, non-inflammatory and bilateral disease of the cornea, which is most frequently diagnosed after adolescence [1]. However, it may also be observed with a less incidence in pre-adolescent children [2]. Here, it has a faster progression and may impair vision if remained untreated [3-7], leaving corneal transplantation as the only option [8]. Due to the importance of good visual acuity in crucial learning years, and to prevent extensively invasive procedures, an efficient and rapid treatment is needed in pediatric keratoconus.

Progressive keratoconus treatment primarily consists of crosslinking (CXL), which enhances inter-collagen bonds and stabilizes the cornea [9]. Moreover, CXL has been shown to have a small but positive effect on corrected distance visual acuity (CDVA) in children with an improvement of about 1 line in the follow-up [9-13] and a mildly regulating effect on corneal shape [14]. Due to the aggressive nature of keratoconus in children, our treatment protocol includes CXL for every child [2, 15]. In a second step, treatment should focus on the improvement of corrected and uncorrected visual acuity, with several options at hand, including intrastromal corneal ring segment (ICRS) implantation. ICRS flattens the conus and regulates the surface of the cornea, hence improving CDVA and uncorrected distance visual acuity (UDVA), also in keratoconus [16]. A paper recently published by our study group reported visual and refractive outcomes of ICRS combined with CXL in pediatrics [15]. Based on these results, our impression was that ICRS insertion in pediatrics might have an enhanced effect compared to adults. 

Human collagen fibers undergo progressive changes with age. In vitro studies on corneas have demonstrated a gradual loss in its elasticity [17, 18] and, at the molecular level, an increase in the cross-sectional areas of each molecule, believed to be due to an increase in the extent of cross-linking between the collagen molecules [19]. These age-related biochemical and biomechanical variances in corneas could play a role in clinical presentation of keratoconus and in corneal response to treatment regarding patient age, i.e. the quickly progressive nature of keratoconus in pediatric patients might indicate an enhanced therapeutic effect. 

To better understand the effect of ICRS and CXL in pediatric patients, we decided to retrospectively analyze the postoperative corneal response and its dependence. This information is especially important in guiding clinicians to effectively treat keratoconus in children and may shed some light on variations of postoperative corneal remodeling. 

## MATERIALS and METHODS

This retrospective matched case-control series included 26 eyes of young patients (≤18 years) as the case group and 26 eyes of adults as control with similar keratoconus parameters. All patients underwent the same treatment as sequential ICRS implantation followed by CXL treatment with an interval of 4 weeks. All procedures were performed at the Beirut Eye Specialist Hospital, Lebanon, between December 2011 and February 2016. Keratoconus was diagnosed based on a combination of the anterior and posterior corneal surface topography, keratometric readings and the corneal pachymetry (Wavelight Allegro Oculyzer, Alcon Laboratories, Inc., Fort Worth, TX) [20, 21]. Disease progression was defined as an increase in maximum keratometry of 1.00 diopter (D) or more in 1 year and was an indication for CXL treatment only in the adult group. Patients younger than 15 years were treated without waiting for signs of disease progression. Indication for surgical treatment was advanced keratoconus with decreased CDVA and hard contact lens intolerance or the desire to remain spectacle free. Implantation of one ring segment was performed to improve mainly CDVA, whereas implantation of two ring segments was performed to improve mainly UDVA. Thickness of ICRS was dependent on the magnitude of refractive error to be treated. The decision on the number, type, thickness and place of insertion was based on our topography guided nomogram for ICRS insertion and theoretical analysis of corneal remodeling after ICRS implantation [22]. Hard contact lens wear was discontinued 3 weeks before the operation.

All patients or their legal parties in the pediatric group signed an informed consent before the operation. All surgeries were performed by the same surgeon (EJ). The study was approved by the Institutional Review Board. All the steps were in accordance with the Declaration of Helsinki. 

Selection of matched cases between the two groups: Cases were matched according to keratoconus parameters (similar keratoconus grading, keratometry, topographic pattern) and treatment (the same ring type, size, arc length and number of ring segments and placement of the ring in similar locations relative to the cone). From a pool of 188 eyes of 106 adult patients, 26 eyes were selected to be compared with the eyes of the pediatric patients. Types of ICRS were Keraring SI6, (Mediphacos, Belo Horizonte, Brazil) and Intacs SK (Addition Technology Inc., Des Plaines, Illinois, The USA) with thickness ranging from 210μm to 450μm. 

Pentacam topographic values including maximum keratometry (Kmax), flat keratometry (Kflat), steep keratometry (Ksteep), the thinnest central corneal thickness (CCT), index of surface variance (ISV), index of height asymmetry (IHA), index of vertical asymmetry (IVA), index of height decentration (IHD), keratoconus index (KI), the smallest sagittal curvature radius (Rmin), center keratoconus index and topographic keratoconus classification (TKC) preoperatively and 1 year after CXL were recorded. Further parameters studied were sphere, cylinder, spherical equivalent, UDVA and CDVA. Changes between preoperative and postoperative values were calculated and compared between the two groups for all the parameters. 

Surgical procedure

Details of the surgical procedure of ICRS and CXL have been published earlier [15]. In brief, ICRS insertion was performed in all cases under topical anesthesia using a femtosecond laser (Intralase FS60; Abbott Medical Optics, Santa Ana, CA). The minimum corneal thickness below the ring was aimed to be at least 100μm and peripheral pachymetry was used to determine the depth of ring insertion. Type of ICRS, thickness, arc length and number of rings were defined according to our protocol for ICRS insertion [21]. CXL was performed according to the standard epithelium-off protocol of Wollensak et al. [23] and was performed one month after ICRS insertion. 

Statistical analysis was performed using SPSS (version 22.0, SPSS, Inc., IL, The USA) software. Independent samples T-Test was used to compare means between the groups. 

## RESULTS

Preoperative comparability

Preoperative parameters for each group are detailed in Table 1 and preoperative TKC are shown in Table 2. In the children group 26 eyes of 19 patients were included with a mean ± standard deviation (SD) age of 14.4±2.7 years (ranged 9-18; 14 males, 5 females), whereas the adult group included 26 eyes of 24 patients with a mean ± SD of 28.5±5.2 years (ranged 21-36; 13 males, 11 females). Mean ± SD of ring thickness was 356±75μm and the average number of rings was 1.35 for both groups. Twenty eyes received 1 Keraring segment, 14 eyes 1 Intacs segment and 18 eyes 2 Intacs segments, equally distributed among the age groups. Except for IVA (*P=0.048*), there was no significant difference between the groups for any preoperative parameter (Table 1).

**Table 1.** Preoperative Characteristics and Comparative Strength Between the Groups for all the Parameters

Parameter (mean ± SD)	Children Age, Y: 14±2.7	Adults Age, Y: 28±5.2	*P-value*
ICRS type	Keraring: n=10 Intacs: n=16	Keraring: n=10 Intacs: n=16	
ICRS thickness	356±75	356±75	1.00
Number of ICRS (micrometer)	1.35±0.48	1.35±0.48	1.00
UDVA (logMAR)	0.84±0.48	0.72±0.44	0.482
CDVA (logMAR)	0.32±0.26	0.26±0.19	0.366
Sphere (D)	-6.16±3.69	-6.67± 4.52	0.677
Cylinder (D)	3.75±1.65	3.51±1.65	0.626
SE (D)	-4.29±3.65	-4.92±4.51	0.605
Kmax (D)	57.20±8.41	56.89±5.26	0.872
Ksteep (D)	51.52±5.93	50.51± 4.65	0.497
Kflat (D)	47.19±5.86	46.79±4.43	0.787
ISV	92±40	103±27	0.291
IHA	28.5±19.0	27.7±20.1	0.889
IVA	0.90 ± 0.39	1.13±0.41	0.048*
IHD	0.090 ±0.063	0.101 ±0.044	0.488
KI	1.24±0.19	1.27±0.10	0.499
Rmin	5.98 ±0.76	5.95±0.55	0.886
CKI	1.08±0.05	1.06±0.05	0.319

*Except for the index of vertical asymmetry (IVA), no parameter had any statistical significant difference. Abbreviations: CDVA: corrected-distance visual acuity; logMAR: the Logarithm of the Minimum Angle of Resolution; CKI: central keratoconus index; D: diopter; ICRS: intracorneal ring segments; IHA: index of height asymmetry; IHD: index of height decentration; ISV: index of surface variance; K: keratometry readings; Kmax: maximum keratometry reading; KI: keratoconus index; Rmin: the smallest sagittal curvature radius; SE: spherical equivalence; UDVA: uncorrected distance visual acuity; SD: standard deviation; Y: years; n: number; logMAR: The Logarithm of the Minimum Angle of Resolution. P˂0.05 in bold.

**Table 2:** Preoperative Automatic Topographic Keratoconus Classification (TKC) of Eyes

	Abnormal	1	1-2	2	2-3	3	3-4	4
Children	0	0	3	14	1	4	3	1
Adults	0	0	1	7	5	10	3	0

**Table 3:** Pre- and Postoperative Values for all Parameters Including the Percentage of Improvement

	Children				Adults			
Parameter Mean ± SD	Preop	Postop	p	%	Preop	Postop	p	%
UDVA (logMAR)	0.84±0.48	0.41±0.29	**0.001**	45%	0.72±0.44	0.39±0.37	**<0.007**	36.8%
CDVA (logMAR)	0.32±0.26	0.15±0.12	**0.001**	40.4%	0.26±0.19	0.15±0.13	**0.001**	42.2%
S (D)	-6.16±3.69	-3.21±3.24	**<0.001**	54.8%	-6.67± 4.52	-2.71±2.77	**<0.001**	56.5%
C (D)	3.75±1.65	2.43±1.53	**0.004**	30.6%	3.51±1.65	2.43±1.67	**0.02**	30.8%
SE (D)	-4.29±3.65	-2.00±2.90	**<0.001**	58.7%	-4.92±4.51	-1.49±2.46	**<0.001**	70.6%
Kmax (D)	57.20±8.41	55.48±7.40	**<0.004**	2.7%	56.89±5.26	55.13±5.85	**0.007**	3.1%
Ksteep (D)	51.52±5.93	48.47±4.61	**<0.001**	5.7%	50.51± 4.65	48.41±4.06	**<0.001**	4.1%
Kflat (D)	47.19±5.86	44.50 ±4.35	**<0.001**	5.4%	46.79±4.43	45.01±4.15	**<0.001**	3.7%
ISV	92±40	75±32	**<0.001**	17.6%	103±27	84.24±30.12	**<0.001**	18.6%
IHA	28.5±19.0	20.1±13.9	**0.03**	29.5%	27.7±20.1	22.3±16.3	0.19	19.5%
IVA	0.90 ± 0.39	0.71 ±0.34	**0.002**	15.3%	1.13±0.41	0.89±0.41	**0.002**	19.0%
IHD	0.090 ±0.063	0.058±0.052	**<0.001**	34.2%	0.101 ±0.044	0.065±0.040	**<0.001**	33.5%
KI	1.24±0.19	1.15±0.16	**<0.001**	7.7%	1.27±0.10	1.18±0.11	**<0.001**	7.1%
Rmin	5.98 ±0.76	6.14±0.72	**0.01**	-2.9%	5.95±0.55	6.17±0.641	**0.006**	-3.6%
CKI	1.08±0.05	1.10±0.05	**0.001**	-2.3%	1.06±0.05	1.09±0.05	**<0.001**	-2.8%

For both groups all the parameters improved significantly after the operation. Abbreviations: P: P- value; Preop: preoperative; Postop: postoperative; %: percentage of improvement; CDVA: corrected-distance visual acuity; logMAR: the Logarithm of the Minimum Angle of Resolution; CKI: central keratoconus index; S: Spherical power; C: Cylinderical power; Ksteep: Steep Keratometry; Kflat: Falat Keratometry; D: diopter; IHA: index of height asymmetry;IHD: index of height decentration; ISV: index of surface variance; IVA: index of vertical asymmetry; Kmax: maximum keratometry reading; KI: keratoconus index; Rmin: the smallest sagittal curvature radius; SE: spherical equivalence; UDVA: uncorrected distance visual acuity; SD: Standard Deviation. P˂0.05 in bold.

Postoperative results 

Table 3 shows the pre- and postoperative values and their difference for all parameters in both groups. All parameters showed a statistical significant improvement. 

Comparison of improvement between the groups

**Figure 1:**
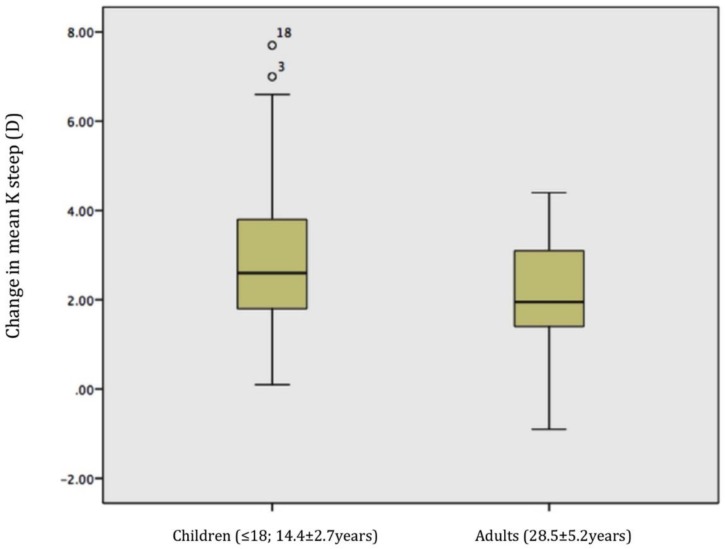
Postoperative change in mean steep keratometry reading (Ksteep) in diopters (D) according to patient age. In the children group Ksteep flattened by 3.05 D, whereas in the adult group Ksteep flattened by 2.10D. This difference was statistically significant (P=0.036). This remained if percentage of change was calculated. Mean ± standard deviation of age in each group is shown

**Figure 2:**
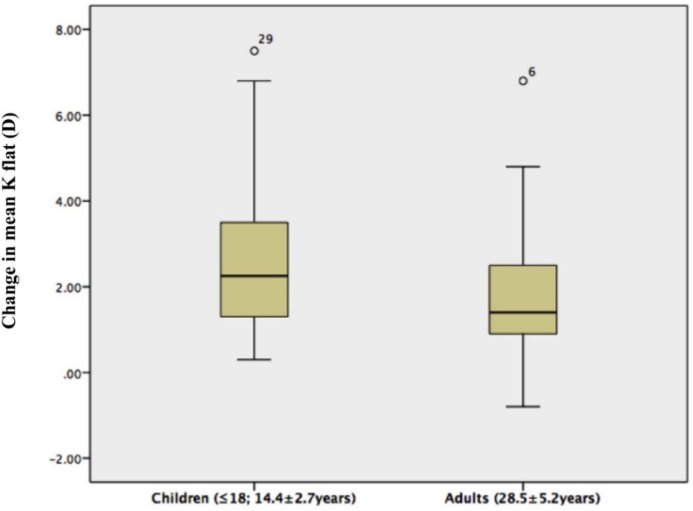
Postoperative change in mean flat keratometry reading (Kflat) in diopters (D) according to patient age. In the children group Kflat flattened by 2.70D, whereas in the adult group Kflat flattened by 1.78D. This difference had a borderline statistical significance (P=0.081). This effect remained if percentage of change was calculated. Mean ± standard deviation of age in each group is shown.

Furthermore, for every parameter the difference between the pre- and postoperative value was compared (Table 4). We noticed that the children group had a significantly more pronounced improvement in Ksteep and a trend to significance in Kflat compared to the adults group. Moreover, for these 2 values, significance remained if percentage of improvement instead of value difference was compared (Figures 1 and 2). Improvements in UDVA and CDVA were more pronounced in children (4.3 and 1.7 ETDRS lines, respectively), compared to adults (3.3 and 1.2 ETDRS lines, respectively), without a statistically significant difference. 

Moreover, there was no statistically significant difference regarding improvement of topographic indices or refractive parameters between the groups. 

**Table 4:** Differences Between Pre- and Postoperative Values

Parameter	Pre- to Postop. Difference, Children	Pre- to Postop. Difference, Adults	P	
UDVA (logMAR)	-0.43	-0.33	0.391	
CDVA (logMAR)	-0.17	-0.12	0.334	
S (D)	2.96	3.96	0.260	
C (D)	-1.31	-1.08	0.656	
SE (D)	2.3	3.43	0.214	
Kmax (D)	-1.72	-1.76	0.964	
Ksteep (D)	-3.05	-2.10	0.036*	
Kflat (D)	-2.7	-1.78	0.081	
ISV	-17	-18	0.754	
IHA	-8.4	-5.5	0.603	
IVA	-0.18	-0.23	0.539	
IHD	-0.033	-0.036	0.702	
KI	-0.10	-0.092	0.734	
Rmin	0.15	0.22	0.462	
CKI	0.02	0.03	0.500	

P: p-value; Pre: preoperative; Postop: postoperative; CDVA: corrected-distance visual acuity; logMAR: the Logarithm of the Minimum Angle of Resolution; CKI: central keratoconus index; D: diopter; S: Spherical power; C: Cylinderical power; Ksteep: Steep Keratometry; Kflat: Falat Keratometry; IHA: index of height asymmetry;IHD: index of height decentration; ISV: index of surface variance; UDVA: uncorrected distance visual acuity; IVA: index of vertical asymmetry; KI: keratoconus index; Rmin: the smallest sagittal curvature radius; SE: spherical equivalence; UDVA: uncorrected distance visual acuity. P˂0.05 in bold.

Safety and efficacy indices 

The safety index (mean postoperative CDVA [decimal] / mean preoperative CDVA [decimal]) [24] were 1.48 and 1.29 for groups 1 and 2, respectively. The efficacy index (mean postoperative UDVA [decimal] /mean preoperative CDVA [decimal]) [24] were 0.79 and 0.75 for groups 1 and 2, respectively.

## DISCUSSION

Recently, our research study group demonstrated the effectiveness and safety of combined ICRS-CXL for the treatment of KCN in the pediatric age group [15]. Our clinical impression was that the ring effect is more accentuated in pediatric patients compared to similar keratoconus characteristics in adults. 

Age variances of biomechanical and biochemical properties of corneal collagen fibers were attributed to a continuous collagen fiber remodeling [16, 17]. Hence, these age-related changes combined with our clinical observation may propose that corneal responses to ICRS implantation in pediatric patients is different to adults. In our study, the effects on clinical and topographic parameters of ICRS implantation followed by CXL in progressive keratoconus were evaluated and compared between the two matched groups. In addition to the type, thickness and number of ICRS and the surgical method, which were all identical, initial topographic and clinical parameters showed a very strong comparability. Of all parameters, only IVA was significantly different between the groups before the operation, with a higher value in the adult population. 

At one year after the procedure, the children group had a significantly higher improvement in Ksteep than the adult group (3.05D versus 2.10D; P=0.036; see Figure 1). A similar effect was observed in Kflat, with 2.7D of improvement in children compared to 1.78D in adults, without a statistically significance difference (*P=0.081)*. Also, it implied to improvement percentage. Concerning functional parameters, it is notable that UDVA improved in children by 4.3 ETDRS lines compared to 3.3 ETDRS lines in adults. Besides, CDVA improved by 1.7 ETDRS lines in children compared to 1.2 ETDRS lines in adults. These differences, however, showed no statistical significance. Lack of statistical significance might be due to a high variance of values in the children group, with some very remarkable improvements in some very young patients. As such, a 13-year old child improved by 10 ETDRS lines in CDVA compared to a maximum improvement of 4 ETDRS lines in adults. As shown in Figure 1, the two distinguishable improvements in Ksteep belonged to two children aged 10 and 12 years.

We believe that the effects of age on the K-readings are not by chance, but rather represent a more dynamic corneal response in children than adults after intervention. The postulated effect is not strong, as there was no significant difference in any of the 7 keratoconus indices between the groups. 

The relatively small age effect may be explained by a high age cut-off. In fact, the cut-off age was chosen arbitrarily, but lowering it to 14 years did not yield further significant differences between the age groups (except for a borderline significance of P=0.08 in Rmin improvement), keeping in mind that the overall number of eyes dropped to 24. 

The present paper presented a large set of data on the effect of ICRS combined with CXL (refer to Table 3). At one year postoperatively, there is a significant improvement in UCVA, BCVA, all refractive parameters as well as in nearly all pentacam topographic indices (except IHA in the adult group), supporting this procedure in improving keratoconus [25-28].

The main limitation of our study was the number of eyes of children included. Several trends have been demonstrated, such as the difference in functional improvement, these can be only confirmed with a higher number of patients. 

Furthermore, our follow-up was short to assess postoperative complications, such as migration or infection of rings, which might happen in the long term. In a previous study with similar surgical intervention and up to 4 years follow-up, 1 of 17 eyes developed a ring migration at 2 years [15].

The present study benefits from a thorough selection of matched cases adult patients, thus minimizing the differences between the groups. However, further prospective studies are needed to evaluate the separate effects of ICRS and CXL, as well as confounding factors such as eye rubbing and allergic keratoconjunctivitis. 

## DISCUSSION

Our study demonstrated a more pronounced effect of corneal response in children (≤18 years) than adults after combined ICRS and CXL for progressive keratoconus. This effect is mild and needs to be verified in further studies. 

## DISCLOSURE

Sylvain El-Khoury and Youssef Abdelmassih contributed equally in this research. Ethical issues have been completely observed by the authors. All named authors meet the International Committee of Medical Journal Editors (ICMJE) criteria for authorship of this manuscript, take responsibility for the integrity of the work as a whole, and have given final approval for the version to be published. No conflict of interest has been presented. Funding/Support: None. 
